# Effect of Electroacupuncture versus Sham Electroacupuncture in Patients with Knee Osteoarthritis: A Pilot Randomized Controlled Trial

**DOI:** 10.1155/2020/1686952

**Published:** 2020-07-30

**Authors:** Qi Wang, Hui Lv, Zhao-Tian Sun, Jian-Feng Tu, Yong-Wei Feng, Tian-Qi Wang, Cun-Zhi Liu

**Affiliations:** ^1^Department of Acupuncture and Moxibustion, Beijing Hospital of Traditional Chinese Medicine, Capital Medical University, Beijing, China; ^2^Department of Acupuncture and Moxibustion, HuGuoSi Hospital of Traditional Chinese Medicine Affiliated with Beijing University of Chinese Medicine and Pharmacology, Beijing, China; ^3^School of Acupuncture-Moxibustion and Tuina, Beijing University of Chinese Medicine, Chaoyang District, Beijing, China

## Abstract

**Objective:**

To explore the feasibility of evaluating the effectiveness and safety of electroacupuncture versus sham electroacupuncture for patients with knee osteoarthritis (KOA).

**Method:**

A pilot randomized controlled trial was conducted at a teaching hospital in Beijing. A total of 30 patients with KOA (Kellgren grade II or III) were randomly allocated to an eight-week treatment of either electroacupuncture or sham electroacupuncture. Patients and outcome assessors were blinded to group allocation. The primary outcome was the proportion of responders achieving at least 1.14 seconds decrease in the Timed Up and Go Test (TUG) at week eight compared with baseline. The secondary outcomes included the knee range of motion, the knee extensor and flexor muscle strength, Lequesne index, 9-step stair-climb test (9-SCT), and TUG.

**Results:**

Of 30 patients allocated to two groups, 27 (90%) completed the study. The proportion of responders was 53.3% (8 of 15) for electroacupuncture group and 26.7% (4 of 15) for sham electroacupuncture group by the intention-to-treat analysis (*P* = 0.264). There was no statistically significant difference in TUG between the two groups at eight weeks (*P* = 0.856). The compliance rate measured according to patients who conformed to the protocol and had received treatments ≥20 times was 93.3% (28 of 30). The dropout rate was 20% (3 of 15). Adverse effects were not reported in the study.

**Conclusion:**

Our research demonstrated that further evaluation of the effectiveness of electroacupuncture versus sham electroacupuncture was feasible and safe for patients with KOA. Whether or not the electroacupuncture can improve the physical functions of knee joint, expand the knee range of motion, and increase the extensor and flexor muscle strength more significantly than sham electroacupuncture, future studies can be designed with larger sample size, randomization design and less biases. This trial is registered with NCT03366363.

## 1. Introduction

Knee osteoarthritis (KOA) is one of the most common degenerative joint diseases. KOA mainly affects the older people. The prevalence rate of female is higher than that of male [1]. The risk factors for onset of KOA include obesity, older age, female gender, intensive physical activity, previous knee injury, presence of Heberden's nodes, and increased bone mineral density [2]. Pain and stiffness are the most typical symptoms of KOA, which can lead to restricted knee range of motion and extensor and flexor muscle weakness. Furthermore, the quality of life can be impaired [3].

Nonoperative treatments are suitable for mild-to-moderate KOA. However, surgical treatments are generally needed for severe KOA [4]. Nonoperative treatments are used to relieve pain, expand range of joint movement, increase muscle strength around the joint, and improve joint physical functions and quality of life [5]. Nonsurgical treatments of KOA consist of pharmacological treatments and nonpharmacological treatments. Some serious adverse effects often occur with pharmacological treatment, such as digestive symptoms [6]. Therefore, nonpharmacological treatment plays an important role in therapeutic options of KOA. Nonpharmacological treatment includes biomechanical interventions, exercise, self-management and education, and weight management. Acupuncture and transcutaneous electrical nerve stimulation also belong to nonpharmacological treatment modalities [7]. Electroacupuncture, as a nonpharmacological treatment, has been proved to be a safe therapy as well [8].

A meta-analysis of randomized controlled trials demonstrated that electroacupuncture had more significant advantages for relieving pain and improving the physical functions than pharmacological treatment and manual acupuncture [8]. It has been suggested that the mechanism of electroacupuncture treatment is attributed to endogenous release of *β*-endorphin resulting in analgesia [9]. Furthermore, the relief of pain may improve physical functions. Electroacupuncture treatment is also able to improve gait performance significantly. Through gait analysis after electroacupuncture treatment, it is observed that electroacupuncture treatment can relieve pain and make patients walk with a higher speed and step length as well as providing better body weight transfer through increasing the dynamic joint ranges of motion and joint moments [10].

Currently, patient-reported measures such as the Western Ontario and McMaster Universities arthritis index (WOMAC) and Lequesne index are often used in evaluating the physical functions of knee joint. However, performance-based tests such as the knee range of motion, knee extensor and flexor muscle strength, 9-step stair-climb test (9-SCT), and Timed Up and Go Test (TUG) are rarely applied in the trials involving electroacupuncture treatment. In this study, in order to evaluate the physical functions of knee joint more objectively, the physical functions are evaluated using both patient-reported measure and performance-based measures as described above. Furthermore, we do not select three-dimensional gait analysis because of its high cost and low compliance rate in patients. Three-dimensional gait analysis is not suitable to be used for a larger sample size trial.

Our primary hypothesis was that electroacupuncture and sham electroacupuncture would have different effects in improving the physical functions of knee joint, expanding the knee range of motion, and increasing the extensor and flexor muscle strength in patients with chronic KOA. The results of this trial will help inform the design of a future, large randomized controlled trial.

## 2. Patients and Methods

This study was a randomized, blinded, sham acupuncture-controlled pilot trial approved by the research ethical committee of Beijing Hospital of Traditional Chinese Medicine Affiliated to Capital Medical University (2017BL-077-01). An approval for patient's safety was also obtained from the research ethical committee. This research was carried out at Beijing Hospital of Traditional Chinese Medicine. The patients we recruited were from the primary clinical study protocol that has been registered at ClinicalTrials.gov (NCT03366363) [11]. All patients provided written informed consent to participate in the study. This study was performed in accordance with the principles of the Declaration of Helsinki.

We recruited patients from the Beijing metropolitan area through advertisements in the community, newspapers, WeChat (a social media platform), and clinics between January 2018 and December 2018. The inclusion criteria were an age of 45–75 years; both genders; unilateral or bilateral KOA participants diagnosed according to the American College of Rheumatology criteria [12]; informed consent signed; KOA classified as grade II or III based on Kellgren-Lawrence criteria [13] within 6 months; knee pain with an average severity of 4 or more out of 10 on a numeric rating scale (NRS) in the last week; duration of unilateral or bilateral knee pain for the last six months. The exclusion criteria were history of knee surgery for the most painful knee or waiting for knee surgery for either knee; knee pain caused by joint bodies, severe effusion of joint cavity, or infection; other diseases causing pain in the knee, such as malignant tumor, autoimmune disease, trauma, fracture, gout, lumbar, and sacral disease; serious acute or chronic organic diseases or mental disorders or blood coagulation disorders; history of knee arthroscopy in the past year; intra-articular injection in the past six months; acupuncture allergy or acupuncture fear; having a cardiac pacemaker; pregnancy or breastfeeding. Patients were also excluded if they had received acupuncture or massage treatment or physical therapy or medication for KOA or participated in other clinical trials in the past three months.

Eligible patients were randomly assigned to the electroacupuncture group or sham electroacupuncture group in a 1 : 1 ratio through a central web-based randomization tool. Computer aided randomization sequence was generated by an independent statistician using the software SAS 9.3 (SAS Institute, Cary, NC, USA) according to a random block size of 6. The independent statistician was not involved in treating the patients or measuring their outcomes and statistical analysis. The sequence was integrated into the software (Beijing Guide Technology Co, Ltd). The research coordinator gained a random number by uploading the name of the patient to a computer system.

Patients were blinded to group assignment. The investigator who assessed the outcomes and the statistician who made the statistical analysis were not also informed about the group allocation. Blinding of the acupuncturists was not carried out because of the nature of the intervention. Different research tasks were carried out by different people.

## 3. Intervention

Acupuncturists were state-licensed and qualified for at least ten years of clinical experience. Patients of each group received three sessions weekly for eight weeks (one session every other day). The duration of every session was 30 minutes. Both knees of patients with bilateral osteoarthritis were needled, whereas, for patients with unilateral osteoarthritis, the affected knee was needled. Disposable, sterile steel needles (25 mm × 0.25 mm or 40 mm × 0.25 mm; Huatuo, Suzhou, China) were used for acupuncture treatment. Before acupuncture manipulation, the skin of acupoints was disinfected with alcohol. Other therapies affecting symptoms were not permitted, such as knee surgery, knee arthroscopy, intra-articular injection, massage, and physical therapy and medication for KOA. During the study period, if the patients did not tolerate knee pain, a temporary oral painkiller (Tylenol, Shanghai Johnson & Johnson Pharmaceuticals, Ltd) was given to them.

### 3.1. Electroacupuncture Group

Electroacupuncture treatment was implemented according to semistandardised principle. A total of eight acupoints were selected including five obligatory acupoints and three adjunct acupoints according to the study protocol [11]. All acupoints are localized according to the WHO Standard Acupuncture Point Locations in the Western Pacific Region [14].

The obligatory acupoints included *ST35*, *EX-LE5*, *GB33*, and *LR8* and an *ashi* point (the point where the patient felt most pain). Adjunct acupoints were selected from 22 acupoints (*ST32*, *ST34*, *EX-LE2*, *ST36*, *ST40*, *SP10*, *KI10*, *SP9*, *LR7*, *SP6*, *KI3*, *LR3*, *SP4*, *BL39*, *BL40*, *BL57*, *BL60*, *GB31*, *GB34*, *GB36*, *GB39*, and *GB41*) according to the meridians in which pain occurred. *ST32*, *ST34*, *EX-LE2*, *ST36,* and *ST40* belonged to stomach meridian (the anterior aspect of the affected knee joint). *SP10*, *KI10*, *SP9*, *LR7*, *SP6*, *KI3*, *LR3,* and *SP4* belonged to three-yin meridian (the medial aspect of the affected knee joint). *BL39*, *BL40*, *BL57,* and *BL60* belonged to bladder meridian (the posterior aspect of the affected knee joint). *GB31*, *GB34*, *GB36*, *GB39,* and *GB41* belonged to gallbladder meridian (the lateral aspect of the affected knee joint). Three adjunct acupoints were chosen by acupuncturists among all the affected meridians.

Acupuncturists were instructed to achieve *de qi* (a composite of sensations including soreness, numbness, distention, and heaviness) through manipulations of twirling, lifting, and thrusting. The needles were stimulated manually for at least 10 seconds. Afterwards, paired electrodes from an electroacupuncture apparatus (HANS-200A acupoint nerve stimulator, Nanjing Jisheng Medical Co, Ltd) were attached to the needles with alligator clips at LR8 and GB33 and another two adjunct acupoints by the research coordinator according to the study protocol [11]. The four acupoints with electrodes were located in the side of the affected knee joint. The stimulus intensity increased until the skin around the acupoints shivered slightly without pain. The wave was set as 2/100 Hz (2 Hz and 100 Hz switched automatically every three seconds) with a duration of 30 min [11].

### 3.2. Sham Electroacupuncture Group

The needles were inserted vertically into eight nonacupoints about 2-3 mm deep without manipulation by acupuncturists. The sensations of *de qi* were not achieved in the sham acupuncture group. Nonacupoints were away from the traditional acupoints or meridians. The locations of eight nonacupoints were defined according to the relevant literature and predefined protocol [11]. Procedures, electrode placements, and other treatment settings were the same as in the electroacupuncture group but with no electricity output.

## 4. Outcome Measures

All patients were evaluated by the investigator at baseline and at weeks 8, 16, and 26.

The primary outcome was the proportion of responders achieving at least 1.14 seconds decrease in TUG at week 8 compared with baseline [15]. The TUG test was recommended as a useful and reliable test of physical function for assessing patients with KOA [16]. The TUG test was used to measure the time a patient took to stand up from a chair (approx. 46 cm seat height with 65 cm arm rest height), walk 3 m at a regular pace, turn around, and return to sit down on the chair. The faster time of the two tests was scored [16].

The secondary outcomes included knee range of motion, knee extensor and flexor muscle strength, Lequesne index, 9-SCT, and TUG. The Lequesne index was applied as patient-reported measure, and knee range of motion, knee extensor, and flexor muscle strength, as well as 9-SCT, were employed as performance-based tests.

Lequesne index is used to evaluate severity and activity of KOA. It includes 11 questions regarding pain, walking ability, and daily activities. Total scores ranged from 0 to 24 points. Higher scores represent worse physical function. Two to three points represent a mild dysfunction. Twenty-four points as the highest score denote the heaviest dysfunction. Seven points or less represent an acceptable dysfunction [17]. A change of approximately two units for a baseline score of 9–11.5 may be clinically meaningful [18].

The measurements of the knee range of motion include active maximum flexion knee range of motion (AROM) and passive maximum flexion knee range of motion (PROM) with a long armed universal goniometer (Shanghai C-MART TOOLS limited company, D0025) according to the neutral position measurement method [19, 20]. It was a valid and reliable measure in patients with knee restrictions [21]. For patients with bilateral KOA, the more painful knee was accessed throughout the entire study [22]. Patients maintained their prone position with extended knee. When AROM was measured, each patient was asked to flex one knee to bring the heel of the evaluated leg as close as possible to the buttock while the other knee remained straight. When PROM was measured, the investigator helped the patient to bend his/her knee further by exerting force on the crus of patients until patient's knee could not bend. The fulcrum of the goniometer was placed on the lateral epicondyle of femur of the evaluated leg, while the stationary arm and mobile arm were in line with the greater trochanter and the lateral malleolus, respectively [23]. The AROM and PROM of the tested limb were measured for three tests. Mean values of the AROM or PROM were calculated for each patient.

The manual muscle testing (MMT) was performed to measure the hamstring and quadriceps muscle strength according to the principles of manual muscle testing and manual muscle test grading system [24, 25]. The MMT is a useful and convenient tool for evaluating the muscle strength [26]. For patients with bilateral KOA, the more painful leg was accessed throughout the entire study [22]. For quadriceps muscle strength measurements, the patients were told to sit on the edge of a diagnostic couch. Their legs dropped naturally and their knees flexed about 90 degrees. The hand of the investigator was placed against the anterior aspect of their ankles. The investigator applied resistance to resist the knee extension movement of patients. Patients were asked to complete the knee extension movement as much as possible. The quadriceps muscle strength of the tested limb was measured for three tests. Mean values of the muscle strength grade were calculated for each patient.

For hamstring muscle strength measurements, the patients maintained a prone position on a diagnostic couch and their knees were flexed about 45 degrees. The hand of the investigator was placed against the posterior aspect of the leg. The investigator applied resistance to resist the knee flexion movement of patients. Patients were asked to complete the knee flexion movement as much as possible. The hamstring muscle strength of the tested limb was measured for three tests. Mean values of the muscle strength grade were calculated for each patient.

A muscle strength scale was used to rate the muscle strength from 0 to 5 according to the muscle performance against resistance [27]. To facilitate data analysis, the muscle scale was converted to an expanded scale of 0 to 12 [25].

The 9-SCT was also recommended as a valid performance-based test of physical function for assessing patients with KOA [16]. The 9-SCT was used for measuring the total time patients required to go up and down nine stairs (20 cm height each step) at their normal speed. If necessary, patients could use auxiliary walking tools such as crutches, which must be recorded in the trial [16].

### 4.1. Sample Size

This trial was a pilot study to prove the feasibility for further clinical trials. The exploratory nature of the trial did not necessarily require a formal sample size calculation. Thus, a total of 30 subjects (each group of 15 cases) were determined as the sample size according to clinical experience and previous similar study [28]. According to the outcomes of the study, we will calculate the sample size of further clinical trials.

### 4.2. Statistical Analysis

Statistical analysis was performed using SPSS 17.0 Software (SPSS Inc, Chicago, IL, USA) by intention-to-treat analysis. The primary outcome was also analysed by per-protocol analysis for sensitivity analysis. The last observation carried forward method was applied to impute missing data. Statistical significance was considered at a two-tailed *P* < 0.05. Descriptive statistics were presented as mean ± standard deviation (SD) or number (%). To compare the equivalence of the baseline characteristics, independent *t*-tests and Pearson chi-square were used accordingly. Paired *t*-tests were used to evaluate the changes before and after treatment in electroacupuncture group and sham electroacupuncture group. The comparison between the two groups was performed using independent *t*-tests. We used the SPSS 17.0 Software to verify normal distribution of the variables. If the distribution of the variables did not belong to the normal distribution, the differences within groups and between the two groups were assessed by Wilcoxon rank sum test.

## 5. Results

**Figure 1:**
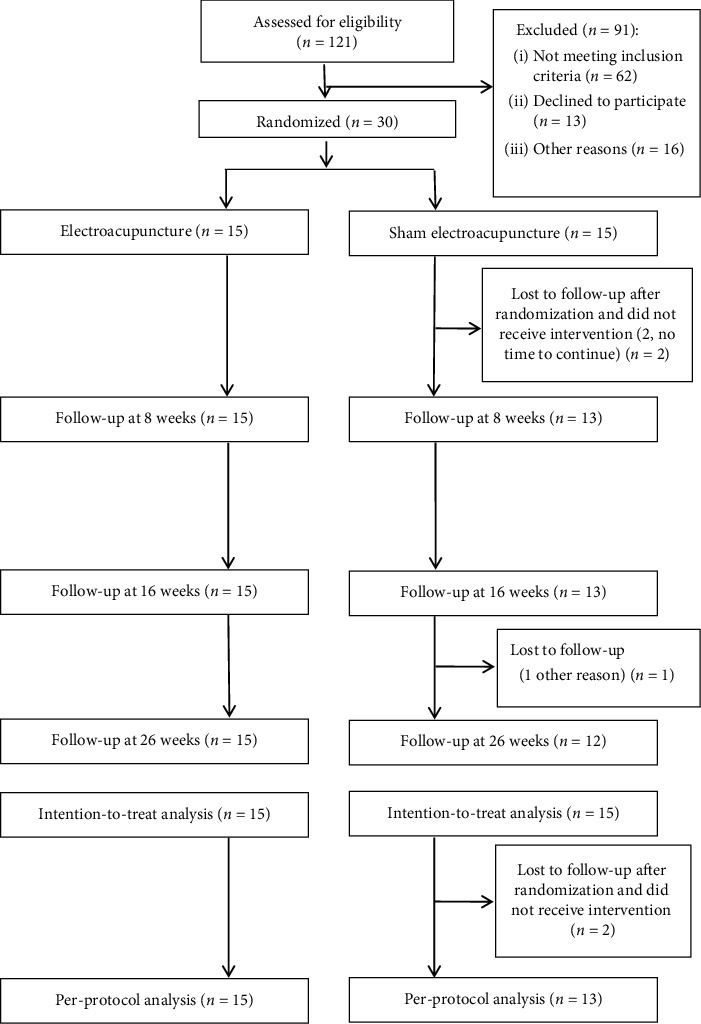
Flowchart of the trial: patients of each group received three sessions weekly for eight weeks (one session every other day).

One hundred and twenty-one patients were recruited for the trial between January 2018 and December 2018. Ninety-one patients were excluded because they failed to satisfy the inclusion criteria, they had any of the exclusion criteria, they declined to participate in the trial, or they could not take part in the trial because of other reasons. Thirty patients were eligible for the study and divided into two groups. Three patients dropped out in sham electroacupuncture group (20% dropout rate). Of the three patients who dropped out of the trial, two patients in sham electroacupuncture group were lost to followup right after the beginning of the treatment and one patient in sham electroacupuncture group was lost to followup at week 26 (Figure 1). There were no statistically significant differences in the baseline characteristics between the two groups (Table 1). Temporary oral painkillers were not taken by any of the patients. Adverse effects after treatment were not reported in the study.

### 5.1. Primary Outcome

The primary outcome is shown in Table 2 according to the intention-to-treat analysis. The proportion of responders in electroacupuncture group at week eight was twice as much as that in sham electroacupuncture group with no statistically significant difference (electroacupuncture group: 53.3% (*n* = 8); sham electroacupuncture group: 26.7% (*n* = 4); *P* = 0.264) (Table 3). There was no statistically significant difference between the two groups for proportion of responders according to the per-protocol analysis (*P* = 0.276) (Table 3).

### 5.2. Secondary Outcomes

Table 2 and Table 4 summarize the secondary outcomes according to the intention-to-treat analysis. There were significant improvements from baseline to week eight in electroacupuncture group in all of the secondary outcomes except hamstring muscle strength (AROM: *P*=0.024; PROM: *P*=0.029; quadriceps muscle strength: *P*=0.012; Lequesne index: *P* ≤ 0.001; TUG: *P*=0.001; 9-SCT: *P*=0.001; hamstring muscle strength: *P*=0.238) (Table 4). However, the statistically significant differences between baseline and week eight in sham electroacupuncture group were not observed in all of the secondary outcomes except Lequesne index and TUG (AROM: *P*=0.600; PROM: *P*=0.285; quadriceps muscle strength: *P*=0.484; hamstring muscle strength: *P*=0.582; 9-SCT: *P*=0.883; Lequesne index: *P*=0.007; TUG: *P*=0.020) (Table 4).

In addition, there were no statistically significant differences in AROM, PROM, quadriceps muscle strength, hamstring muscle strength, Lequesne index, TUG, and 9-SCT between the two groups over time (Table 2). The differences in the change from baseline to week eight between the two groups were not evident in the secondary outcomes except for AROM, PROM, and 9-SCT (AROM: *P*=0.027; PROM: *P*=0.012; quadriceps muscle strength: *P*=0.539; hamstring muscle strength: *P*=0.582; Lequesne index: *P*=0.211; TUG: *P*=0.162; 9-SCT: *P*=0.041) (Table 2).

## 6. Discussion

In the trail, the patients with KOA who received the electroacupuncture treatment experienced better physical functions after treatment compared with baseline, while the improvements of functions did not occur in the patients who received the sham electroacupuncture treatment except for Lequesne index and TUG. Nevertheless, at all of time points, the therapeutic effectiveness of electroacupuncture was not superior to that of sham electroacupuncture in the secondary outcomes. In addition, the magnitude of the improvements from baseline to week eight in AROM, PROM, and 9-SCT were more evident in electroacupuncture group than in sham electroacupuncture group. Similar results were not observed in the other outcomes. However, for Lequesne index, the changes from baseline and eight weeks may be clinically meaningful for the two groups because of achieving at least 2 units of decrease. All in all, stronger effectiveness was not observed for the electroacupuncture treatment in improving physical functions, expanding the knee range of motion, and increasing the extensor and flexor muscle strength for patients with KOA after eight weeks' treatment. The conclusion may be not comparable with that of the peer study which evaluates effectiveness of electroacupuncture and manual acupuncture versus sham acupuncture for KOA, because the outcome measures of our trial are different from those of the peer study. The peer study applied patient-reported measures to assess the effectiveness of interventions in patients with KOA. However, in our study, we applied TUG (performance-based measure) as the primary outcome to assess the physical functions in the knee joint of patients with KOA. In addition, the data of the peer study is not yet published [11].

At present, some trials proved that electroacupuncture is capable of alleviating the pain and improving the physical functions of KOA patients apparently with few adverse effects [29–31]. Among these trials, two trials showed that high-intensity electroacupuncture alleviated pain of KOA patients more effectively than low-intensity electroacupuncture [30, 31]. In another trial, patients with lower KOA Kellgren grades could gain more benefits from electroacupuncture in pain relief and physical functions improvements [29]. An overview of systematic reviews suggested that the effectiveness of electroacupuncture prevailed over that of western medicine with respect to Lequesne index [32]. Furthermore, through three-dimensional motion analysis, electroacupuncture was verified to increase the speed of ascending and descending stairs of KOA patients in a trial [33]. Besides the clinical trials, several experimental researches indicated the effectiveness of electroacupuncture regarding the mechanism of electroacupuncture for KOA. For instance, electroacupuncture was also able to inhibit the expression of IL-1*β* by activating the CB2 receptor and then relieved pain in the mouse model of KOA [34]. Moreover, electroacupuncture retarded cartilage degradation through pain relief and potentiation of muscle function [35].

By comparison with the previous studies, our study was different in intervention methods and outcome measures. In addition, our study was a pilot study with small sample size. So the conclusion of our study may be different from those of the previous studies.

In most cases, previous studies applied subjective scales to assess the effectiveness of electroacupuncture in patients with KOA. But in our trial, both patient-reported measure and performance-based measures were applied in the measurement of physical functions in the knee joint of patients with KOA. Through the two assessment methods, physical functions of knee joint were evaluated more objectively. Meanwhile, the subjective bias was also reduced [36]. With regard to the blind method, sham electroacupuncture was designed to decrease the subjective effects of different treatments on patients. More importantly, the adverse effects were not found in our trial. Besides, in our pilot study, we needed to explore the feasibility of further study with larger sample size. In the trial, 24.8% (30 of 121) of volunteers screened were recruited during a four-month period, which is similar with the recruitment rate of another study [22]. The successful recruitment rate reflects that a large number of patients with KOA are willing to participate in the trial. Compliance rate measured according to patients who conformed to the protocol and had received treatments ≥20 times was 93.3% (28 of 30). The dropout rate was 20% (3 of 15). The trial suggested that the research process could be implemented with minimal difficulty. As a consequence, the safety and patient adherence were reliable in the trial. Above all, future clinical trials could be feasible and safe.

Although our trial had advantage in practice and feasibility, some limitations in the trial should be taken into consideration. First, because of our small sample size, it was easy to produce type II error resulting in reduction of electroacupuncture efficacy. On the basis of this trial, the sample size of future study was calculated with 80% power and *α* = 0.05. In order to detect a difference between electroacupuncture group with 53.3% response rate and sham electroacupuncture with 26.7% response rate, at least 67 patients were required in each group with the consideration of a 20% dropout rate for the future study. Second, superficial penetration of sham electroacupuncture also played a certain effect in acupuncture treatment. A systematic review about sham- (placebo-) controlled randomized acupuncture trials identified that the more verum and sham acupuncture share the same dermatomes, the closer the clinical outcomes will be [37]. In addition, the patients with KOA may benefit from the positive expectancy for pain relief. A functional neuroimaging study suggested that the positive expectancy can produce analgesic effects by neural mechanisms [38]. Third, we did not test the awareness of patients about the treatment assignment, which was necessary in the future studies. Fourth, because missing data in sham electroacupuncture group was imputed with the use of the last observation carried forward method, the assumed data was identical from beginning to the end of the trial. Furthermore, a false conclusion might be created if the assumed data and actual data were different in fact [39]. So, in our trial, there was an unexpected improvement in sham electroacupuncture group due to the bias of the results. Patient-acupuncturist interactions in sham electroacupuncture group could also result in an unexpected clinical improvement on the basis of an update of a systematic review [40]. Fifth, in the trial, acupuncturists used the semistandardised principle to select treatment prescription in the light of the acupuncturists' experiences, which might cause selection and efficacy bias. The bias of outcomes could also result from the investigator when assessed outcomes.

## 7. Conclusions

In conclusion, the study indicates that further evaluation of the effectiveness of electroacupuncture versus sham electroacupuncture was feasible and safe for patients with KOA. Whether or not the electroacupuncture can improve the physical functions of knee joint, expand the knee range of motion, and increase the extensor and flexor muscle strength more significantly than sham electroacupuncture, future studies can be designed with larger sample size, randomization design, and less biases.

## Figures and Tables

**Table 1:** Baseline characteristics of intention-to-treat population.

Characteristics	Electroacupuncture (*n* = 15)	Sham electroacupuncture (*n* = 15)	*P*
Female, no. (%)	13 (86.7)	11 (73.3)	0.361
Male, no. (%)	2 (13.3)	4 (26.7)	0.361
Age, mean (SD), years	63.5 (8.4)	65.2 (7.6)	0.572
BMI, mean (SD), kg/m^2^	24.2 (2.8)	26.0 (2.9)	0.110
Duration, mean (SD), months	61.3 (47.6)	68.2 (62.4)	0.737
Kellgren criteria			
Kellgren 2, no. (%)	7 (46.7)	7 (46.7)	1.000
Kellgren 3, no. (%)	8 (53.3)	8 (53.3)	
AROM, mean (SD), °	116.8 (13.3)	115.4 (14.2)	0.775
PROM, mean (SD), °	124.9 (13.6)	124.2 (14.6)	0.887
Quadriceps muscle strength score, mean (SD)	9.9 (1.2)	10.4 (1.5)	0.350
Hamstring muscle strength score, mean (SD)	8.7 (0.9)	9.0 (1.2)	0.395
Lequesne index score, mean (SD)	10.6 (3.2)	10.7 (3.7)	0.958
TUG, mean (SD), s	13.8 (4.0)	12.9 (4.8)	0.594
9-SCT, mean (SD), s	21.3 (12.0)	18.7 (11.6)	0.564

BMI: body mass index; AROM: active maximum flexion knee range of motion; PROM: passive maximum flexion knee range of motion; TUG: Timed Up and Go Test; 9-SCT: 9-step stair-climb test. Significant *P* < 0.05.

**Table 2:** Comparison of variables between the two groups (intention-to-treat).

Variables	Electroacupuncture (*n* = 15)	Sham electroacupuncture (*n* = 15)	*P*
Proportion of responders, no. (%)			
Week 8	8 (53.3%)	4 (26.7%)	0.264

TUG, mean (SD), s			
Week 8	11.4 (3.5)	11.6 (3.9)	0.856
Week 16	10.4 (3.2)	12.6 (5.8)	0.208
Week 26	10.5 (3.4)	12.6 (6.1)	0.239
Change from baseline and 8 weeks	2.4 (2.3)	1.3 (1.9)	0.162

AROM, mean (SD), °			
Week 8	121.2 (13.6)	114.7 (16.1)	0.240
Week 16	118.7 (19.0)	115.4 (17.5)	0.621
Week 26	118.1 (19.4)	112.9 (18.8)	0.468
Change from baseline and 8 weeks	4.4 (6.7)	−0.7 (5.2)	0.027

PROM, mean (SD), °			
Week 8	132.4 (9.3)	122.6 (16.9)	0.059
Week 16	127.8 (17.6)	121.5 (18.4)	0.347
Week 26	127.9 (17.7)	119.6 (20.3)	0.247
Change from baseline and 8 weeks	7.5 (12.0)	−1.5 (5.3)	0.012

Quadriceps muscle strength score, mean (SD) or median (*P*_75_, *P*_25_)			
Week 8	10.6 (1.1)	10.7 (1.5)	0.891
Week 16	10.7 (1.1)	12 (12, 10)	0.233
Week 26	10.8 (1.3)	10.9 (1.4)	0.785
Change from baseline and 8 weeks	0.0 (2.0, 0.0)	0.0 (1.0, 0.0)	0.539

Hamstring muscle strength score, mean (SD)			
Week 8	9.0 (1.3)	9.1 (1.4)	0.786
Week 16	9.1 (1.5)	9.0 (1.3)	0.798
Week 26	9.1 (1.4)	8.9 (1.4)	0.594
Change from baseline and 8 weeks	0.3 (1.0)	0.1 (0.9)	0.582

Lequesne index score, mean (SD)			
Week 8	7.0 (3.9)	8.4 (4.5)	0.383
Week 16	6.4 (4.2)	8.6 (4.8)	0.192
Week 26	6.2 (4.4)	8.7 (4.5)	0.128
Change from baseline and 8 weeks	3.6 (2.7)	2.3 (2.8)	0.211

9-SCT, mean (SD), s			
Week 8	18.0 (8.6)	18.8 (12.0)	0.826
Week 16	16.7 (8.7)	18.7 (12.8)	0.617
Week 26	17.1 (8.4)	19.1 (12.6)	0.617
Change from baseline and 8 weeks	3.3 (5.8)	−0.1 (1.8)	0.041

TUG: Timed Up and Go Test; AROM: active maximum flexion knee range of motion; PROM: passive maximum flexion knee range of motion; 9-SCT: 9-step stair-climb test. Significant *P* < 0.05.

**Table 3:** Comparison of variables between the two groups (per-protocol analysis).

Variables	Electroacupuncture (*n* = 15)	Sham electroacupuncture (*n* = 13)	*P*
Proportion of responders, no. (%)			
Week 8	8 (53.3%)	4 (30.8%)	0.276

Significant *P* < 0.05.

**Table 4:** Mean (SD) comparison of variables in the two groups (intention-to-treat).

	Electroacupuncture (*n* = 15)			Sham electroacupuncture (*n* = 15)		
	Preintervention	Postintervention	*P*	Preintervention	Postintervention	*P*
TUG, s	13.8 (4.0)	11.4 (3.5)	0.001	12.9 (4.8)	11.6 (3.9)	0.020
AROM, °	116.8 (13.3)	121.2 (13.6)	0.024	115.4 (14.2)	114.7 (16.1)	0.600
PROM, °	124.9 (13.6)	132.4 (9.3)	0.029	124.2 (14.6)	122.6 (16.9)	0.285
Quadriceps muscle strength score	9.9 (1.2)	10.6 (1.1)	0.012	10.4 (1.5)	10.7 (1.5)	0.484
Hamstring muscle strength score	8.7 (0.9)	9.0 (1.3)	0.238	9.0 (1.2)	9.1 (1.4)	0.582
Lequesne index score	10.6 (3.2)	7.0 (3.9)	≤0.001	10.7 (3.7)	8.4 (4.5)	0.007
9-SCT, s	21.3 (12.0)	18.0 (8.6)	0.001	18.7 (11.6)	18.8 (12.0)	0.883

TUG: Timed Up and Go Test; AROM: active maximum flexion knee range of motion; PROM: passive maximum flexion knee range of motion; 9-SCT: 9-step stair-climb test. Postintervention: at week eight. Significant *P* < 0.05.

## Data Availability

The data used to support the findings of this study are available from the first author upon request.
